# Integrated vector management targeting *Anopheles darlingi* populations decreases malaria incidence in an unstable transmission area, in the rural Brazilian Amazon

**DOI:** 10.1186/1475-2875-11-351

**Published:** 2012-10-23

**Authors:** Keillen M Martins-Campos, Waléria D Pinheiro, Sheila Vítor-Silva, André M Siqueira, Gisely C Melo, Íria C Rodrigues, Nelson F Fé, MariadasGraçasV Barbosa, Wanderli P Tadei, Caterina Guinovart, Quique Bassat, Pedro L Alonso, Marcus VG Lacerda, Wuelton M Monteiro

**Affiliations:** 1 Fundação de Medicina Tropical Dr. Heitor Vieira Dourado, Av. Pedro Teixeira, 25, Dom Pedro, Manaus, AM, 69040-000, Brazil; 2 Instituto Nacional de Pesquisas da Amazônia, Av. André Araújo, 2936, Aleixo, Manaus, AM, 69060-001, Brazil; 3 Universidade do Estado do Amazonas, Av. Pedro Teixeira, 25, Dom Pedro, Manaus, AM, 69040-000, Brazil; 4 Universidade Federal do Amazonas, Rua Afonso Pena, 1053, Praça 14, Manaus, AM, 69020-170, Brazil; 5 Universidade Nilton Lins, Av. Prof. Nilton Lins, 3259, Parque das Laranjeiras, Manaus, AM, 69058-030, Brazil; 6 Barcelona Centre for International Health Research (CRESIB, Hospital Clínic-Universitat de Barcelona), Rosselló 132, 4°, Barcelona, 08036, Spain

**Keywords:** Malaria, *Anopheles darlingi*, Impregnated bed nets, Indoor residual spraying, Amazon

## Abstract

**Background:**

Studies on vector behaviour should be conducted in order to evaluate the effectiveness of vector control measures on malaria protection in endemic areas of Latin America, where *P. vivax* predominates. This work aims to investigate the fauna of anopheline mosquitoes and verify the impact of integrated vector management in two colonization projects in the Careiro Municipality, Western Brazilian Amazon.

**Methods:**

Four mosquitoes’ captures were carried out from August 2008 to March 2010, with an interval of six months between each collection. Since September 2009 a large programme to reduce the burden of malaria has started in the two communities by distribution of insecticide-treated bed nets (ITN) and intensification of indoor residual spraying (IRS). Human biting rates (HBRs), entomological inoculation rates (EIRs), malaria incidence rate (MIR) and *Plasmodium* carrier’s prevalence were used as outcomes to estimate the impact of the control measures.

**Results:**

A total of 3,189 anophelines were collected, belonging to 13 species. *Anopheles darlingi* was the predominant species in the period (42.6%), followed by *Anopheles albitarsis* (38.4%). *An. darlingi* HBRs showed a notable decreasing trend from the start to the end of the study. Conversely, *An. albitarsis* increased its contribution to overall HBRs throughout the study. For *An. darlingi* there was a significant positive correlation between HBRs and MIR (p = 0.002). *Anopheles albitarsis* HBRs showed a significant negative correlation with the corresponding MIR (p = 0.045). EIR from total anophelines and from *An. darlingi* and *An. albitarsis* presented decreasing patterns in the successive collections. Four species of anophelines (*An. darlingi*, *An. albitarsis, Anopheles braziliensis* and *Anopheles nuneztovari*) were naturally infected with *Plasmodium*, albeit at very low infection rates. There were a decrease in the MIR for both vivax and falciparum malaria and in the prevalence of *Plasmodium vivax* and *Plasmodium falciparum* carriers during the period of study.

**Conclusions:**

There is strong evidence of association between the density of *An. darlingi* and the incidence of malaria in the studies sites, further highlighting the importance of this vector in malaria transmission in this region. *An. darlingi* susceptibility to control using ITN and IRS is likely to be high in the rural settlements studied.

## Background

Malaria remains one of the most important public health problems worldwide, with about 3.3 billion people at risk of contracting the disease and 655,000 deaths estimated annually, mainly in children under five years [1]. In Latin America (LA), approximately 170 million people live at risk of *Plasmodium vivax* and *Plasmodium falciparum* transmission distributed in 21 countries [2]. Approximately 60% of the malaria cases in the Americas are reported from Brazil, where 99.8% of the cases are reported in the Amazon Region [3].

*Anopheles darlingi* is the major vector of malaria in Brazil [4-7], being highly susceptible to *Plasmodium* sp. infection, with highly anthropophilic and endophagic behaviour [7-9]. This species is described in tropical and subtropical regions in Central and South Americas especially in areas of low altitude, preferring large bodies of water in forested areas [10]. The larvae are adapted to the water margins, preferably deep, clean, slightly cloudy, sunny or partially shaded ones, hiding among the vegetation or debris. During rainy seasons they change their behaviour with a preference for breeding at smaller size and depth water bodies [11,12]. Although *An. darlingi* predominates in Brazilian endemic areas, other species of anophelines have also been implicated as malaria vectors in distinct Amazonian settings, namely *Anopheles albitarsis s.l., Anopheles nuneztovari*, *Anopheles triannulatus* and *Anopheles intermedius*[8,13-15]. In some regions of Brazil, these secondary vectors, mainly *An. albitarsis* may invade houses and feed in humans. However, in most of its territory, including the dry north-east and the central areas, this anopheline is exophilic and zoophilic, preferring to attack the cattle [16].

The Malaria Control Brazilian Programme focuses their strategies on the early diagnosis and treatment of infected individuals. However, many efforts in order to control the vectors’ population have been put into practice, such as the use of impregnated bed nets, indoor residual spraying and environmental cleaning [17]. It is important to point out that the effectiveness of such tools has not yet been evaluated in an integrated manner, in part due to the lack of baseline information on the natural behaviour of *An. darlingi* and secondary vectors related to malaria transmission in the Amazon Basin. As malaria vectors are able to adapt to different environmental conditions (more and more frequent in recent occupied settlements in LA), descriptive entomological studies should become a routine practice, supporting vectorial control measures.

In the Southern Amazon Basin, malaria is often concentrated in agricultural settlements and areas of mining activity [18]. Recent occupation of landscape results in higher anopheline population densities [19] which, in association with the arrival of non-immune newcomers, tend to sustain the disease transmission [18,20,21].

Therefore, the aims of this study were to describe the dynamics of the anopheline population in a rural area of recent colonization in the Western Brazilian Amazon, and secondarily to correlate the mosquitoes’ density variation with the incidence of clinical malaria in this area after implementation of integrated control measures focusing on the vector.

## Methods

### Study sites

**Figure 1:**
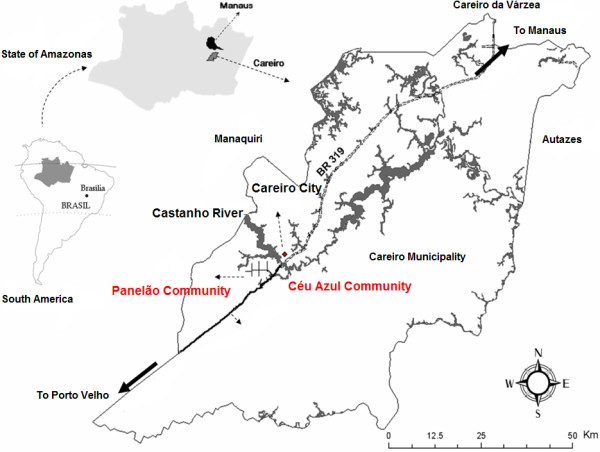
Location of the Careiro Municipality, highlighting the areas of study.

The Municipality of Careiro is located in the central region of the Amazonas State (Western Brazilian Amazon) (03°06’ S; 60°01’ W), 112 km from the capital of the state, Manaus (Figure 1). The estimated population in 2010 was 32,734 people, mainly living in rural areas (71.2%). The main economical activity is related to agriculture (cassava, fruits, vegetables, rice, and sugar cane), cattle breeding, fish-farming, and forestry. These activities have resulted in a decrease in vegetation cover and habitat fragmentation. The remaining vegetal cover is primarily composed of dense macrothermic ombrophilous forest. The climate according to Köppen classification is Af (super-humid equatorial), with the rainy season occurring from November to April, mean pluviometric precipitations above 2,000 mm *per annum*, average temperatures ranging from 26°C to 30°C, and relative humidity between 85 and 90%. The relief of the municipality can be considered plane, at an altitude never exceeding 100 meters above the sea level [22].

Two areas of recent occupation devoted to agriculture were chosen (Panelão and Céu Azul Communities) (Figure 1), with a total population of 736 persons (census performed immediately before the beginning of the study). Water for drinking comes from rainwater reservoirs or creeks. Garbage collection and sanitation are absent. Two health agents in each community are responsible for health care. Both those two agricultural settlements were established by the National Institute for Colonization and Agrarian Reform (INCRA), which primarily aims to rescue the productive potential of the family agriculture.

### Anopheline sampling

Four mosquitoes’ captures were carried out from August 2008 to March 2010, with a 6-month-interval, comprising two collections in the dry season (August) and two in the rainy (March) seasons. The collections were performed in 10 households, randomly selected having in consideration the spatial distribution of the areas, being one in each of the six ancillary roads in the community of Panelão, a more densely populated and more fragmented area, and four in the community of Céu Azul, in which the household are located in both sides of a main road that connects Manaus with Porto Velho. Simultaneous 4-hour captures were performed indoors and outdoors from 6 to 10 pm by harmonized and trained technicians, due to previous 12- h collections studies in several localities of the Brazilian Amazon demonstrating the mosquitoes’ peaks of host-seeking activity [6,23]. Outdoor collection sites were at 20 metres distances from the households. Six technicians worked in each group, divided in pairs, one person capturing mosquitoes indoors and the other outdoors. Each capturer worked for 1 hr and then rested for the next hour. Mosquitoes that landed on the capturers’ legs were captured with Castro aspirators. Immediately the insects were placed in appropriate containers identified with date, time, location and collector. The light conditions and closing of doors and windows followed the same as of the normal behavior of the residents. The same households were visited in each of the four six-monthly collections. In both studied communities, captures were performed predominantly in the same house over the entire study period. Temperature and rainfall data were obtained from satellite imaging from the National Institute of Meteorology.

The identification of anophelines was performed using Consoli and Lourenço-de-Oliveira [24] and Faran and Linthicum [25] keys. The morphological differences of the members of the nuneztovari and oswaldoi complexes, with exception of *Anopheles deaneorum* were not defined. Therefore, *An. nuneztovari*, *Anopheles oswaldoi* and *An. albitarsis* in this work refer to the species *sensu latu.* Margalef’s diversity index [26] was used for determining species diversity in the different capturing periods. This index was calculated as D = (S – 1)/ln N, where *S* = number of species and *N* = number of individuals.

After identification, mosquitoes were stored in 2 mL Eppendorf tubes with silica gel. In each tube, up to 10 mosquitoes from the same species collected at the same time, environment, and collector were stored. The tubes were kept in a room protected from high temperatures and humidity until the PCR technique for *Plasmodium* sp. detection was made, after the conclusion the last cross-sectional time point.

### Natural infection of mosquitoes by *Plasmodium* sp

For total DNA extraction from a pool of head and thorax of 10 mosquitoes, 300 μL of 5% w/v Chelex 100 in water were added to each tube. The insects were smashed with a pestle and placed at 100°C for 10 min and spinned at 14,000 g for 15 min. Two hundred microlitres of supernatant were transferred into a new tube and incubated to -20°C. We used 0.5 μL as template in a 10 μL PCR reaction. DNA was amplified in an Applied Biosystems 7500 Fast System® using primers [27] and TaqMan fluorescence labeled probes for real-time PCR [28] as previously described.

### Vector control measures

In September 2009, the local Malaria Control Programme improved vector control actions, such as the free distribution of insecticide-treated bed nets, window screening and intensification of indoor residual spraying (cypermethrin).

### Epidemiological study

Malaria incidence rates (MIR) were obtained from a longitudinal study carried out in the two communities in the period. Passive case detection based on microscopic examination of Giemsa stained thick blood smears (TBSs) was performed for every episode of fever among the members of the cohort.

Concurrently to the entomological investigation, four cross-sectional surveys of the cohorts under study were conducted in order to determine the prevalence of malaria through active case detection. The laboratory diagnosis of malarial infection was based on nested PCR amplification of a species-specific segment of the 18 S rRNA gene of human malaria parasites in blood samples collected in filter paper. The extraction of total DNA from filter paper was performed using the QIAamp® DNA Blood Mini Kit (Qiagen®, USA), according to the manufacturer’s protocol. DNA was amplified in an Applied Biosystems 7500 Fast System® using primers [27] and TaqMan fluorescence labeled probes for real-time PCR [28] as previously described.

### Data analysis

The malaria transmission potential of mosquitoes was determined by estimating the human biting rates (HBR) and entomological inoculation rates (EIR) for each collection in each location. HBR was scored as the average hourly number of mosquitoes captured per person. The EIR was estimated by multiplying the HBR and infection rates (IR). The IR is the proportion of positive pools in which Plasmodium sp. was detected by PCR.

Malaria prevalence calculations were based on the number of cases diagnosed by PCR and the number of people found during the four cross-sectional surveys. Incidence of malaria obtained from the longitudinal study was used to calculate the incidence rates (cases/1,000 persons/year) taking into account the cases communicated from each site of anopheline collection and the population follow-up time. Malarial incidence rates (MIRs) were determined for each anopheline collection site using the population of the site and the number of cases of malaria registered one month and a half before and after the collection’s date.

Pearson’s test performed in SPSS 16.0 software was used to verify the correlation between HBRs (for total anophelines and for An. darlingi and An. albitarsis separately) and the malarial incidence rates (cases/1,000 persons/year).

HBR, EIR, MIR and malaria prevalence were used as outcomes to estimate the impact of the control measures established in the middle of the period of study.

### Ethics procedures

Human surveys and entomological investigation were approved by the National Ethics Review Committee (protocol number 15197). Mosquitoes’ collection using the above mentioned capture methodology was preceded by ethical clearance. All malaria cases detected in the longitudinal or cross-sectional studies were treated according to the Brazilian Ministry of Health’s National guidelines.

## Results

**Figure 2:**
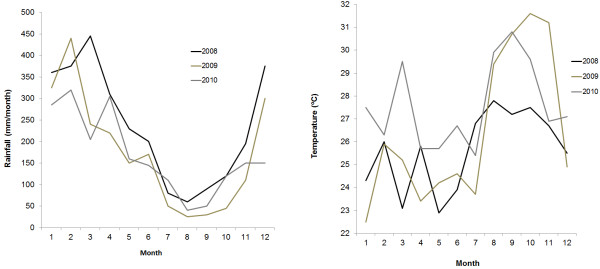
Rainfall (A) and temperature (B) parameters, in the studied localities during the period of the study (2008-2010).

Rainfall and temperature were homogeneous throughout the period of the study, showing the expected typical seasonality, with increased rainfall during the first six months of the year, followed by increased mean temperatures in the second semester of the years (Figure 2).

A total of 3,189 anophelines were collected from the households under entomological surveillance at the 4 different timepoints. Anopheline mosquitoes belonged to 13 different species (Table 1). *Anopheles darlingi* was the predominant species in the period, representing 42.6% of the total of anophelines collected, followed nearly by *An. albitarsis*, with 38.4%. In all the collections there was a predominance of mosquitoes in the peridomicilliary environment (77.1%). *Anopheles darlingi* and *An. albitarsis*, the two major species, predominated outdoors, where 83.3% and 65.5% were collected, respectively. All the other species also prevailed in this environment (Additional file 1). Margalef’s index ranged from 0.59 to 1.54, with values slightly higher in collections carried out in the peridomiciliary area. This index showed a decreasing trend in the diversity of anopheline species along the period of study (Additional file 1).

**Table 1:** Anopheline species collected in the Panelão and Castanho Sítio Communities during the four cross-sectional studies

**Anopheline species**	**Number (%)**
**Subgenus*Nyssorhynchus*** (Blanchard, 1902)	
*Anopheles albitarsis* (Lynch-Arribálzaga, 1878)	1224 (38.4)
*Anopheles argyritarsis* (Robineau-Desvoidy, 1827)	105 (3.3)
*Anopheles braziliensis* (Chagas, 1907)	371 (11.6)
*Anopheles darlingi* (Root, 1926)	1357 (42.6)
*Anopheles deaneorum* (Rosa-Freitas, 1989)	27 (0.8)
*Anopheles evansae* (Brèthes, 1926)	2 (0.06)
*Anopheles nuneztovari* (Gabaldón, 1940)	63 (2.0)
*Anopheles oswaldoi* (Peryassú, 1922)	7 (0.2)
*Anopheles triannulatus* (Neiva & Pinto, 1922)	23 (0.7)
**Subgenus*Anopheles*** (Meigen, 1902)	
*Anopheles mediopunctatus* (Theobald, 1903)	4 (0.1)
*Anopheles mattogrossensis* (Lutz & Neiva, 1911)	3 (0.1)
*Anopheles peryassui* (Dyar & Knab, 1908)	1 (0.03)
*Anopheles punctimacula* (Dyar & Knab, 1906)	2 (0.06)
**Total**	**3189 (100)**

The HBRs calculated for all anophelines and for *An. darlingi* and *An. albitarsis* by collection and specific locality are shown in Additional file 2. Overall HBRs did not show an important variation in the different localities of collection along the period. The first collection showed HBRs ranging from 3.1 to 16.3, the second collection 0.4 to 3.6, the third collection 0.3 to 14.3 and the last collection 0.8 to 21.0. HBRs calculated for *An. darlingi* showed a notable decreasing trend from the start to the end of the study. The first collection showed *An. darlingi* maximum HBRs of 15.6, falling to 2.8 and 4.0 in the last collections. On the other hand, *An. albitarsis* increased its contribution to overall HBRs along the time. The first collection showed *An. albitarsis* maximum HBRs of 2.0, subsequently peaking at 21.0 during the fourth collection. In the end of the study, in some localities the overall HBRs were almost exclusively due to *An. albitarsis*. The increase in the HBRs for this species was most notable in the localities of the Céu Azul Community. The other species showed very low HBRs and great fluctuations along the collections.

**Figure 3:**
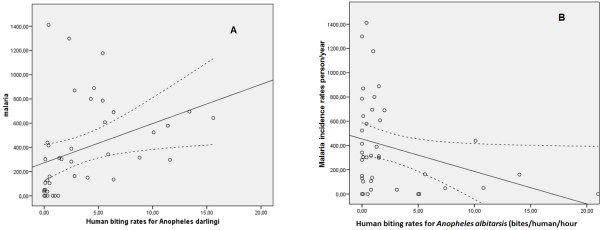
**Correlation between malaria incidence rates (MIRs) and human biting rates (HBRs) for *Anopheles darlingi *(A) and *Anopheles albitarsis *(B), with 95% confidence intervals (dashed lines), respectively.**

Figure 3 summarizes the correlation between the two major vector specific HBRs and MIRs in the corresponding period and locality. For *An. darlingi* there seemed to be a significant positive correlation between HBRs and MIR (p = 0.002). This positive correlation was maintained for *An. darlingi* HBRs obtained outdoors (p = 0.001), but the correlation was lost indoors (p = 0.107). Conversely, overall *An. albitarsis* HBRs showed a significant negative correlation with the corresponding MIR (p = 0.045) (Figure 3).

**Figure 4:**
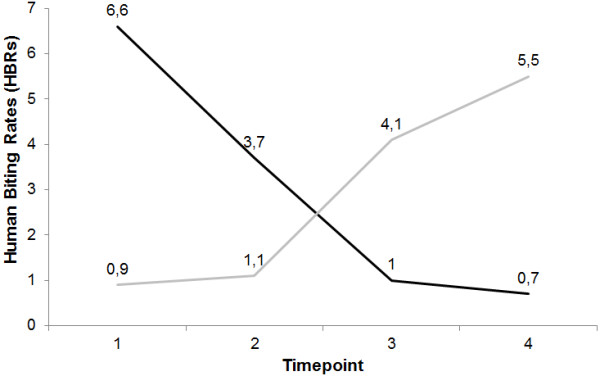
**Patterns of human biting rates (HBRs) for *Anopheles darlingi *(black line) and *Anopheles albitarsis *(gray line) presented by timepoint of collection**.

HBRs for all the mosquitoes ranged from 9.2 in the first collection to 7.5 in the fourth. Figure 4 shows that *An. darlingi* HBRs showed a decreasing pattern, especially after the second collection, when control measures were intensified. *An. albitarsis* HBRs behaved in a contrary manner, with an increasing trend along the period. This trends were confirmed when we analysed the HBRs calculated for indoor and outdoor environments separately, for both species.

EIR from total anophelines and for *An. darlingi* and *An. albitarsis* presented decreasing patterns in the successive collections. This decrease was particularly notable for the two major collected anophelines, which demonstrated near to zero EIRs after the beginning of control measures intensification.

Four species of anophelines were found naturally infected with *Plasmodium* sp*.* In the first collection one pool of *An. albitarsis* was positive for *Plasmodium vivax*. The second collection showed two pools of *An. darlingi* and one pool of *An. albitarsis* positive for *P. vivax*. In the third collection, one pool of *An. braziliensis* was positive for *P. vivax* and one pool of *An. nuneztovari* was positive for *P. vivax/Plasmodium falciparum* mixed infection. One pool of *An. braziliensis* of the fourth collection was positive for *P. vivax/P. falciparum* mixed infection. Four infected pools came from outdoor and three from indoor captures (in this case only for *An. darlingi* and *An. albitarsis*).

**Figure 5:**
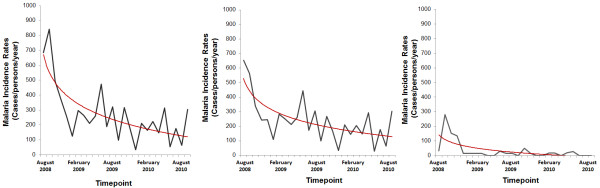
Decreasing patterns of overall malaria incidence rates (A), vivax malaria (B) and falciparum malaria (C) (trend line in red) throughout the 18 months of the study in the study areas.

**Figure 6:**
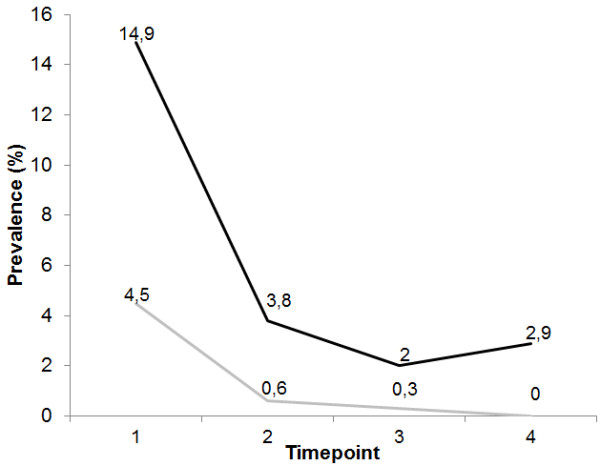
**Decreasing pattern of prevalence of*Plasmodium vivax*(black line) and*Plasmodium falciparum*(gray line) carriers, detected by peripheral blood PCR positivity, in the four cross-sectional evaluations performed in the study areas.**

Figures 5 and 6 show a clear decrease in the MIR for both vivax and falciparum malaria and in the prevalence of *P. vivax* and *P. falciparum* carriers, respectively, during the period of study.

## Discussion

Insecticide-treated nets, indoor residual spray, and the use of biolarvicides are the major vector control tools used in the fight against malaria. When integrated vector management (IVM) strategies have been consistently implemented in African countries, they have successfully controlled malaria transmission [29]. However, IVM has been slowly introduced for malaria control in LA and it has not been sufficiently evaluated in this region [30]. Considering the long-term challenge of malaria eradication, it is essential to increase knowledge on the ecology and behavior of malaria vectors [31,32]. In LA, current methods for vector control are based primarily on the use of long-lasting insecticide treated nets and indoor residual spraying, but there is no available information on the impact that these measures have on suppressing anopheline populations and reducing levels of malaria parasite transmission in recent agrarian reform projects in the Amazon.

Studies focusing on mosquito bionomics may provide solid baseline information to direct malaria control strategies. All the anopheline species collected in this study have been previously reported in the Amazon Region [4,6]. Margalef’s index observed in the period was low, irrespective of the collection environment. *Anopheles darlingi* was the predominant species in the period, followed by *An. albitarsis*. Eleven species showed infrequent human biting habits. *Anopheles darlingi* is considered as the primary malaria vector in Brazil based on its high susceptibility to *Plasmodium* sp., and its anthropophilic and endophagic behaviour [7-9]. In a previous report, 87.8% out of 27,428 *An. darlingi* specimens collected in the Amazon were collected within households, confirming its strong anthropophilic habits [33]. In this study, however, in all collections there was a predominance of mosquitoes in the peri-domiciliary environment, in agreement with collections performed during the construction of the Manaus-Boa Vista Highway [6]. It is not possible to conclude that peri-domiciliary transmission in the two communities occurs more frequently than indoor transmission, but there was evidence of strong association between malaria incidence and *An. darlingi* outdoor HBRs.

In the state of Roraima, Northern Brazilian Amazon, the decrease in distribution and density of anophelines corresponded to a malaria decrease in surrounding areas [34]. In Manaus, a relationship between *An. darlingi* density increases and the number of malaria cases 30 days later was observed [6]. However, few studies have demonstrated a correlation between longitudinal data on malarial incidence with abundance and HBRs for this species, confirming its effective contribution to transmission, as strongly suggested by the present data. In the State of Amapá, Brazil [35], and in French Guiana [36], significant correlations were detected between malaria cases and suspected vector species abundance. As shown in this study, and despite the low diversity of the anopheline fauna, malaria remains in endemic levels at these sites possibly because of the abundance and vectorial competence of the major anophelines. In fact, results from entomological investigations carried out in recent rural Amazon settlements suggest that an intensification of anthropic environmental changes increases the abundance of *An. darlingi*[19], contributing to maintain several malarial foci with high incidence rates in recent colonization places [18-20].

*Anopheles darlingi* showed to be the vector responsible for the transmission of malaria in the communities, as demonstrated by the strong correlation between its HBRs and MIRs. The decrease in malaria incidence during the period of study is likely to be linked to the vector control measures. The other species of anophelines, especially *An. albitarsis*, were not sensible to the control strategies. On the other hand, the latter species increases in abundance along the investigation, correlating negatively with malaria incidence and showing that theirs densities does not have major importance in the transmission of malaria in the communities. Although the abundance and infection by *Plasmodium* sp. in entomological investigations carried out in the Brazilian Amazon [6-9,15,16,37], the present data pointed that the role of *An. albitarsis* as a malaria vector is unclear and needs to be further investigated in this region. In some regions of Brazil, *An. albitarsis* may invade houses and feed in humans. However, in most of its territory, including the dry northeast and the central areas, this anopheline is exophilic and zoophilic, preferring to attack the cattle [16]. It can be speculated that the substitution of *An. darlingi* by *An. albitarsis* was due to the increase in the bovine cattle breeding that occurred in the communities in the recent years, particularly in the Céu Azul Community, diminishing the breeding sites for *An*. *darlingi*, and intensifying attraction of *An. albitarsis* in neighbouring houses.

Four outcomes were used to evaluate the impact of the vector control measures (HBRs, EIRs, MIR and prevalence of *P. vivax* and *P. falciparum* carriers). All these indicators suggested an effectiveness of the use of impregnated bed nets and residual spraying in two rural communities. Although very low infection rates were seen, EIRs decreased throughout the study. HBRs seem to decrease in parallel to the prevalence of *P. vivax* and *P. falciparum* carriers, and with MIR, reinforcing the potential of these vector control approaches in the rural Amazonian areas. A clear limitation of this study that needs to be highlighted is the descriptive methodology employed in this study, without any controlled strategy, which would be considered unethical. Moreover, the use of bed nets was not appropriately evaluated. Unfortunately, there is no systematic information concerning acceptability of these methods in the two communities. In Southern Brazilian Amazon, impregnated mosquito nets were not effective in controlling malaria [38]. In Guatemala and Nicaragua, malaria incidence was significantly lower in populations using insecticide-impregnated bed nets [39,40]. A high proportion of *P. falciparum* was listed between the most important factors for the success of the bed net programme in Ecuador, Colombia and Peru [41]. In the Colombian Amazon, promotion of mosquito net use and impregnation had a benefit as evidenced by a case-control study [42].

## Conclusions

Data presented here represent an important contribution to the knowledge of the epidemiology of malaria transmission and vector control in agricultural settlements in the Amazon, indicating that *An. darlingi* is the vector responsible by the transmission in these sites, and that integrated measures using impregnated bed nets and residual spraying may reduce substantially the man-vector contact and malaria incidence. Fortunately *An. darlingi* susceptibility to control is likely to be high in the rural settlements studied, but a major long-term threat for malaria vector control is the development of insecticide resistance, which stresses the necessity to develop local and systematic evaluation. In malaria endemic areas of Latin America, where *P. vivax* is predominant, studies on vector behaviour should be conducted in order to predict the impact of vector control measures on malaria transmission.

## Competing interests

The authors declare that they have no competing interests.

## Author's contributions

KMMC, WDP, IR, GCM and NFF participated in data collection and laboratory procedures. SVS participated in field-works. MVGL and AMS participated in overall study conception and design, data collection, analysis, interpretation and manuscript preparation. WMM, MGVB, WPT, CG, QB e PLA were involved in data interpretation and manuscript preparation. All authors read and approved the final manuscript.

## Supplementary Material

Additional file 1 Abundance and diversity of the anopheline fauna presented by collection. Click here for file

Additional file 2 **Human biting rates for the total of anophelines, *Anopheles darlingi *and *Anopheles albitarsis *presented by collection and localities.** Click here for file

## Aknowledgements

We acknowledge the collaboration of the local microscopists Juscelino Torres dos Santos, Eliana Lima and Irailton dos Santos. This study was supported by *Conselho Nacional de Desenvolvimento Científico e Tecnológico* (CNPq) (grant number 575788/2008-9) and by *Fundació Cellex* (*P. vivax* Consortium).
