# Supplementary material for: Integrated vector management targeting Anopheles darlingi populations decreases malaria incidence in an unstable transmission area, in the rural Brazilian Amazon
Source: Malar J. 2012 Oct 23;11:351. doi: 10.1186/1475-2875-11-351 (PMC3502175; doi:10.1186/1475-2875-11-351)
Supplement: Additional file 1 — Abundance and diversity of the anopheline fauna presented by collection. [file 1475-2875-11-351-S1.doc]

**Additional file 1.** Abundance and diversity of the anopheline fauna presented by collection.

| **Species** | **1^st^ collection** | |  | **2^nd^ collection** | |  | **3^rd^ collection** | |  | **4^th^ collection** | |  | **All collections** | | **Total (%)** |
| --- | --- | --- | --- | --- | --- | --- | --- | --- | --- | --- | --- | --- | --- | --- | --- |
|  | **Indoor** | **Outdoor** |  | **Indoor** | **Outdoor** |  | **Indoor** | **Outdoor** |  | **Indoor** | **Outdoor** |  | **Indoor (%)** | **Outdoor (%)** |  |
| *An. darlingi* | 116 | 623 |  | 95 | 323 |  | 11 | 107 |  | 4 | 78 |  | 226 | 1131 | **1357** |
| *An. albitarsis* | 31 | 72 |  | 42 | 83 |  | 136 | 246 |  | 213 | 401 |  | 422 | 802 | **1224** |
| *An. braziliensis* | 14 | 80 |  | 2 | 27 |  | 9 | 136 |  | 18 | 85 |  | 43 | 328 | **371** |
| *An. argyritarsis* | 6 | 34 |  | 1 | 64 |  | 0 | 0 |  | 0 | 0 |  | 7 | 98 | **105** |
| *An. nuneztovari* | 6 | 2 |  | 2 | 12 |  | 5 | 3 |  | 7 | 26 |  | 20 | 43 | **63** |
| *An. deaneorum* | 2 | 25 |  | 0 | 0 |  | 0 | 0 |  | 0 | 0 |  | 2 | 25 | **27** |
| *An. triannulatus* | 5 | 4 |  | 3 | 1 |  | 0 | 5 |  | 2 | 3 |  | 10 | 13 | **23** |
| *An. oswaldoi* | 0 | 1 |  | 0 | 4 |  | 0 | 2 |  | 0 | 0 |  | 0 | 7 | **7** |
| *An. mediopunctatus* | 0 | 1 |  | 0 | 3 |  | 0 | 0 |  | 0 | 0 |  | 0 | 4 | **4** |
| *An. matogrossensis* | 0 | 3 |  | 0 | 0 |  | 0 | 0 |  | 0 | 0 |  | 0 | 3 | **3** |
| *An. evansae* | 0 | 1 |  | 0 | 0 |  | 0 | 0 |  | 0 | 1 |  | 0 | 2 | **2** |
| *An. punctimacula* | 0 | 0 |  | 0 | 2 |  | 0 | 0 |  | 0 | 0 |  | 0 | 2 | **2** |
| *An. peryassui* | 0 | 0 |  | 0 | 0 |  | 0 | 0 |  | 0 | 1 |  | 0 | 1 | **1** |
| **Total** | **180** | **846** |  | **145** | **519** |  | **161** | **499** |  | **244** | **595** |  | **730** | **2459** | **3189 (100)** |
| **Margalef’s index** | **1.16** | **1.48** |  | **1.00** | **1.28** |  | **0.59** | **0.80** |  | **0.73** | **0.94** |  | **0.91** | **1.54** |  |
