# Supplementary material for: Integrated vector management targeting Anopheles darlingi populations decreases malaria incidence in an unstable transmission area, in the rural Brazilian Amazon
Source: Malar J. 2012 Oct 23;11:351. doi: 10.1186/1475-2875-11-351 (PMC3502175; doi:10.1186/1475-2875-11-351)
Supplement: Additional file 2 — Human biting rates for the total of anophelines, Anopheles darlingi and Anopheles albitarsis presented by collection and localities. [file 1475-2875-11-351-S2.doc]

**Additional file 2.** Human biting rates for the total of anophelines, *Anopheles darlingi* and *Anopheles albitarsis* presented by collection and localities.

| **Localities** | **1^st^ collection** | | | **2^nd^ collection** | | | **3^rd^ collection** | | | **4^th^ collection** | | |
| --- | --- | --- | --- | --- | --- | --- | --- | --- | --- | --- | --- | --- |
|  | **Overall** | ***An. darlingi*** | ***An. albitarsis*** | **Overall** | ***An. darlingi*** | ***An. albitarsis*** | **Overall** | ***An. darlingi*** | ***An. albitarsis*** | **Overall** | ***An. darlingi*** | ***An. albitarsis*** |
| **Major road** | 3.1 | 2.8 | 0.1 | 3.1 | 2.5 | 0 | 2.1 | 1.6 | 0.1 | 1.1 | 0.1 | 0.8 |
| **Sideroad 1** | 6.9 | 5.4 | 1.0 | 9.6 | 8.8 | 0.8 | 0.6 | 0.5 | 0.1 | 3.0 | 1.4 | 1.5 |
| **Sideroad 2** | 7.4 | 5.6 | 1.6 | 11.6 | 9.6 | 1.5 | 0.3 | 0.1 | 0.1 | 6.9 | 1.3 | 5.0 |
| **Sideroad 3** | 11.9 | 11.4 | 0.4 | 10.1 | 10.1 | 0 | 1.1 | 1.0 | 0.0 | 3.8 | 2.5 | 1.3 |
| **Sideroad 4** | 5.4 | 5.4 | 0.0 | 0.4 | 0.4 | 0 | 2.8 | 2.3 | 0.0 | 0.8 | 0.4 | 0.4 |
| **Sideroad 5** | 16.3 | 15.6 | 0.1 | 6.4 | 5.9 | 0 | 0.5 | 0.3 | 0.0 | 4.0 | 4.0 | 0.0 |
| **Km 114-123** | 8.5 | 6.4 | 2.0 | 8.9 | 6.4 | 0.9 | 1.5 | 0.8 | 0.5 | 6.9 | 0.3 | 5.1 |
| **Km 124-133** | 9.0 | 4.6 | 1.5 | 1.9 | 0.3 | 0.8 | 14.0 | 0.1 | 10.8 | 9.3 | 0.0 | 7.4 |
| **Km 134-143** | 15.1 | 13.4 | 0.9 | 6.0 | 0.0 | 3.1 | 14.0 | 2.8 | 5.6 | 15.4 | 0.3 | 10.1 |
| **Km 144-151** | 11.5 | 4.3 | 1.1 | 2.8 | 0.1 | 0.4 | 14.3 | 0.5 | 14.0 | 21.0 | 0.0 | 21.0 |
